# Use of mTRAQ derivatization reagents on tissues for imaging neurotransmitters by MALDI imaging mass spectrometry: the triple spray method

**DOI:** 10.1007/s00216-019-02052-1

**Published:** 2019-08-22

**Authors:** Toshimasa Ito, Masashi Hiramoto

**Affiliations:** 1 Drug Development Solutions Division, Sekisui Medical Co., Ltd, 2117 Muramatsu, Tokai, Ibaraki, Japan; 2 Analysis and Pharmacokinetics Research Labs., Astellas Pharma Inc., 21 Miyukigaoka, Tsukuba-shi, Ibaraki, Japan

**Keywords:** Relative quantitative comparison, Neurotransmitter, Amino acids, Mass spectrometry imaging, Stable isotope label, Derivatization

## Abstract

During drug development, matrix-assisted laser desorption/ionization (MALDI) imaging mass spectrometry is used for visually elucidating the distribution of substances such as biomarkers, candidate compounds, and metabolites in the tissues. However, it is difficult to make relative comparisons between tissue sections and there are still many challenges. Here, we report a new method of “triple spray” for the comparison of analyte distribution in multiple tissue slices. This method targets amino acids and amines, and it incorporates the application of the internal standard in the on-tissue derivatization step. With further development, it has the potential to alleviate problems caused by the matrix effect. Initially, we measured three serial sections of rat brain to verify the efficacy of this method. In the hypothalamus, where gamma-aminobutyric acid (GABA) is known to be present in high concentration, the GABA levels of the three serial section showed little variation (CV = 1.62%). Subsequently, we compared the GABA level in the brain between stroke-prone spontaneous hypertensive rats (SHRSP) and Wistar-Kyoto (WKY) rats with three individuals each. It showed significant differences between these models at the pre-selected region of interest (*p* < 0.05). Our results show that the triple spray allows for relative comparison among multiple tissue slices with high reproducibility.

**Figure:**
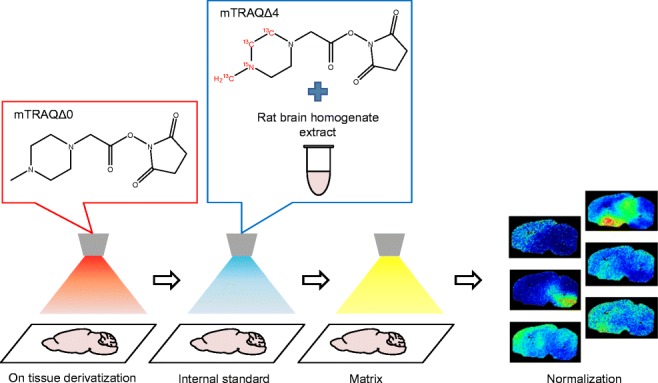
Graphical abstract

Graphical abstract

## Introduction

Matrix-assisted laser desorption/ionization (MALDI) mass spectrometry imaging can visualize the distribution of various biomolecules with high spatial resolution on the order of micrometers, leading to application into many fields, including the use in drug development [1]. In drug development, it is attractive to be able to detect compounds such as biomarkers [2], administered drug, and its metabolite [3] without the use of radioisotope or antibody label. One disadvantage of this method is the reliance on the analyte to be ionizable. However, with increase in the number of studies, the ranges of matrices [4] and derivatization reagents [5] are expanding, allowing for a wider range of analytes to be detected [6, 7]. It is important to be able to quantitatively compare the data for their use in drug development. One of the challenges in MALDI mass spectrometry imaging is the lack of standardized quantification method. The difficulty in standardized quantification is caused by the variation in ion suppression in different tissues, ionization efficiency of analyte, and the formation of uniform matrix crystals on the tissue. To solve the variation caused by ion suppression, various quantitative methods have been studied, of which the method using standard curve being the most widely accepted. For the standard curve, one method conducts direct addition of the standard curve samples onto the tissue [8] and another uses sliced frozen homogenate sample spiked with the analyte standard solution as the standard curve sample [9], which can be effective for tissues with equal ion suppression across the tissue, such as liver tissues. Unfortunately, this method would not be suitable for tissues such as brain, which has varying ion suppression across the tissues. Furthermore, to allow for good quantitative and reproducible data, it is necessary to have equal sensitivity across the whole tissue. This relies on uniform matrix crystal formation especially when handling with very low concentration of analytes in the sample. To solve this problem, an automatic sprayer which can form uniform crystals is necessary, and with the increasing use of MALDI mass spectrometry imaging, various sprayers have been invented and utilized [10]. However, even with the advancement, it has proved to be difficult to obtain uniform matrix crystals, which has resulted on the need for normalization methods to compensate for the variation expressed in the raw experimental data.

From the above, it is necessary to consider both matrix inhomogeneities and ion suppression from endogenous compounds in the tissue in order to generate data that can be meaningfully compared between different tissues and tissues with different morphologies. One method to alleviate this problem is the use of stable isotope–labeled internal standard [11]. For example, normalization of endogenous compounds by stable isotope–labeled internal standard has been reported in a study which obtained mass spectrometry image of GABA, glutamic acid, and acetylcholine in rat brains using DESI-MS by applying the spray solution mixed with the stable isotope of these analytes [12].

Amino acids and neurotransmitting amines are known for their importance as functional molecules in the body, but it has proved to be difficult to measure these compounds. With advancements in on-tissue derivatization methods, more mass spectrometry imaging results have been reported in recent years [13, 14]. On-tissue derivatization improves ionization efficiency and increases the molecular weight of the neurotransmitters which prevents the overlap with MALDI matrix signals often found in the similar mass range to the analyte.

**Fig. 1:**
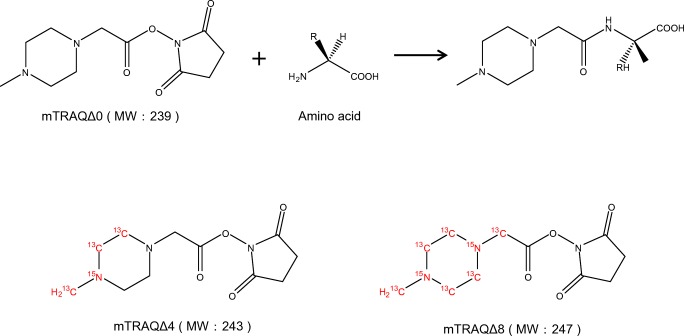
mTRAQ reagent kit is a triplex set of non-isobaric amine labeling reagents. mTRAQΔ4 and mTRAQΔ8 are stable isotopes of mTRAQΔ0

**Fig. 2:**
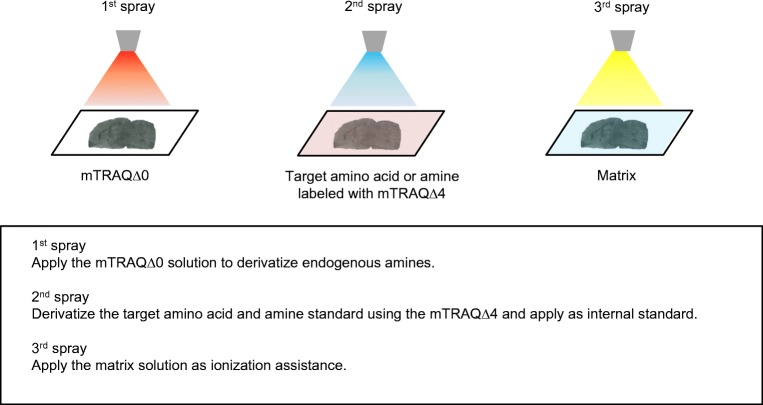
Triple spray requires three steps using an automatic sprayer for the pretreatment

For all of these reasons, we decided to use a stable isotope–labeled derivatization reagent to assist with measuring amino acids and amines from brain tissue. We used mTRAQ reagents, which consist of a group of derivatization agents that react with primary amines and include the non-stable isotope–labeled mTRAQΔ0 and its stable isotope–labeled analogs, mTRAQΔ4 and mTRAQΔ8 [15] (Fig. 1). In combination with the use of these reagents, we came up with the triple spray that is effective for normalizing ion suppression from the tissue (Fig. 2). There are three steps involved in this method. Firstly, mTRAQΔ0 is sprayed onto the tissue section to derivatize the endogenous amino acids and amines. Secondly, the internal standard solution which is labeled with mTRAQΔ4 is sprayed onto the tissue, and lastly, the MALDI matrix solution is sprayed on top. This sample can be measured using imaging mass spectrometry. This triple spray method allows multiple sections to be compared by normalizing the signal of the endogenous amino acids and amines to the isotopically labeled compound without the dependence on MALDI matrix crystal uniformity. In this study, we initially tested the reproducibility of GABA distribution in three serial sections of the rat brain for proof of concept. We selected the brain to test the triple spray because of its heterogeneity which would aid us to clearly show the effectiveness of this method. Furthermore, amino acids and amines are known for their use as neurotransmitters which play an important role in the brain. As for the GABA distribution in the brain, it has been reported that GABA is intensively distributed around the hypothalamus [16] so we used three serial sections of WKY rat brain and evaluated the reproducibility in the concentration around the hypothalamus. Next, we conducted a comparison between different animal models developed for specific diseases by comparing the GABA distribution in rat brain from SHRSP and WKY. It has been reported that the GABA level in the hypothalamus is significantly lower in the SHRSP brain than in WKY [17]; therefore, we looked for similar traits in our data. Furthermore, we comprehensively evaluated a range of amino acids and amine distribution with a normalization method. From these studies, we evaluated the reproducibility, comparability, and the practicality of the triple spray.

## Materials and methods

### Chemicals and reagents

The standard of GABA (purity ≥ 99%), 2,5-dihydroxybenzoic acid (DHB, purity = 98%), and triethylammonium bicarbonate (TEAB) were purchased from Sigma-Aldrich (MO, USA). Trifluoroacetic acid (TFA) was purchased from Wako (Osaka, Japan). mTRAQ reagent kit was purchased from AB Sciex (Foster City, CA). Hydroxylamine was purchased from Tokyo Chemical Industry (Osaka, Japan). Indium tin oxide–coated glass slides were purchased from Matsunami Glass (Osaka, Japan).

### Animals

All studies were conducted in accordance with the Sekisui medical and Astellas policy on care. Age-matched (7-week-old) male WKY and SHRSP rats were obtained from SLC Japan. Rats were housed in a light- and temperature-controlled animal facility and maintained on tap water. WKY and SHRSP rats were fed on F-2 feed and SP feed respectively through their bleeding period. The 8-week-old SHRSP rats were provided with 1% saline as the only liquid source until 9 weeks old. These rats were decapitated at 9 weeks old and the brain was immediately removed and flash-frozen in liquid nitrogen. The brains were stored at − 80 °C until use. Brain tissues were sectioned at 10 μm thickness using a cryostat (CM3050S, Leica Biosystems, Wetzlar, Germany) at − 20 °C.

### Derivatization methods

For on-tissue derivatization with mTRAQΔ0 solution, 20 μL of mTRAQΔ0 reagent was mixed with 80 μL of acetonitrile and 100 μL of water. Next, the internal standard was prepared by mixing 40 μL of mTRAQΔ4 reagent mixed with 120 μL of 200 μg/mL GABA dissolved in TEAB buffer (0.5 M, pH 8.0) and kept at room temperature for 2 h. Excess reagents were quenched with 40 μL of 1.2% hydroxylamine solution. To perform the quantification, firstly, mTRAQΔ0 solutions were applied to brain sections using automatic sprayer (San-ei-tech, Chiba, Japan) and placed into a container with 100 mL of water. The brain sections were then positioned 0.5 mm below the surface and the container was sealed. The container was incubated at 32 °C for 1 h. Secondly, the internal standard solution was applied in the same manner. The spray was set at air pressure of 20 MPa and flow rate of 0.2 μL/min, and approximately 100 μL of solution was sprayed per brain section. Lastly, DHB (30 mg/mL in 70% ethanol, 0.1% TFA) was applied using ImagePrep (Bruker Daltonics, Billerica, MA). The spray was set to apply 1.5 mL in an hour.

### LC-MS/MS

Confirmation of GABA derivatization was conducted using LC-MS/MS. An aliquot (5 μL) of the GABA standard solution and the derivatized GABA standard solution was injected on a SCIEX API 4000 LC-MS/MS system (AB Sciex, Foster City, CA) using an Intrada Amino Acid column (3 μm, 3 mm I.D. × 150 mm L; Imtakt, Kyoto, Japan) at 400 μL/min flow rate with a column oven set at 35 °C. The analysis was conducted with positive ion mode scanning with a gradient separation method using mobile phase A of methanol/100 mM ammonium formate (20/80, v/v) and mobile phase B of methanol/water/formic acid (80/20/0.3, v/v/v). The A/B ratios were set at 0/100 for 1 min, changed to 100/0 from 1 to 5 min, and returned to 0/100 immediately, and held for 1 min, resulting with a total of 6 min run time.

### MALIDI imaging mass spectrometry

All MALDI imaging mass spectrometry was performed using a solariX 9.4 T Fourier transform ion cyclotron resonance mass spectrometer (Bruker Daltonics, Billerica, MA). The data were acquired at a spatial resolution of 200 μm. MALDI mass spectra were acquired at each position using 400 laser shots at a frequency of 1 kHz. These data sets were acquired using continuous accumulation of selected ions (CASI), where the mass selective quadrupole was set to only pass *m*/*z* 150 to 750.

### Data analysis

All ion images were generated using FlexImaging v5.0 (Bruker Daltonics, Billerica, MA) from the raw data with a mass tolerance of ± 0.001 Da. The average spectra generated for the region of interest in the tissue sections were exported from FlexImaging to data analysis v5.0 (Bruker Daltonics, Billerica, MA). FlexImaging was used for image acquisition and encompassing the region of interest. All calculations were made using scaled intensities. Endogenous GABA concentration was evaluated using peak intensity ratios of GABA labeled with mTRAQΔ0 to GABA labeled with mTRAQΔ4 peak in the region of interest.

## Results

The aim of this study was to develop a method which gives quantitative and reproducible result utilizing on-tissue derivatization. Our approach allows a relative comparison of separately prepared sections by utilizing the mass difference in mTRAQΔ0 and mTRAQΔ4. In this study, we focused on quantitatively evaluating GABA because it is an important neurotransmitter and there are many reported cases in the MALDI mass spectrometry imaging field [18, 19]. For the data analysis, peak intensity of endogenous GABA labeled with mTRAQΔ0 was normalized by that of GABA standard solution labeled with mTRAQΔ4 in order to generate the images and values without the factor of ion suppression.

### On-tissue derivatization of amino acids by mTRAQΔ0

**Fig. 3:**
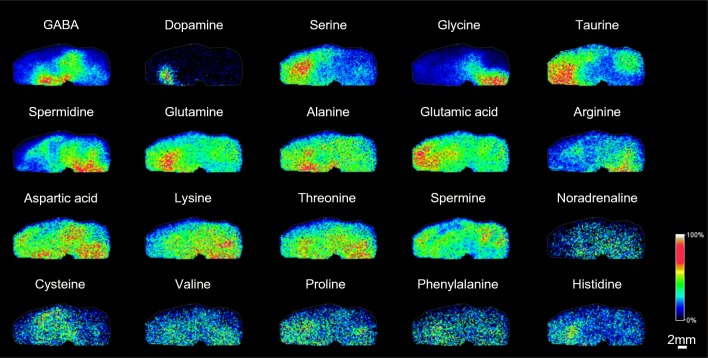
mTRAQΔ0 can derivatize 20 amino acids and amines. These images were obtained before normalization; hence, these distributions still contain the effect of ion suppression from the tissue. Scale bar, 2 mm; spatial resolution = 200 μm

We optimized the conditions for on-tissue derivatization using mTRAQΔ0, with regard to reagent concentration, reaction time, and temperature of mTRAQΔ0. The concentrations of mTRAQ reagents are not open to the public, but one vial of the reagent was the optimum content to derivatize three sections. Furthermore, in order to improve the derivatization efficiency, the use of TEAB buffer is recommended for mTRAQ reaction in liquid phase [20], but for our on-tissue application, the use of TEAB buffer caused decrease in derivatization efficiency. It is desirable for the derivatization to be conducted in a humid environment [21]; thus, the mTRAQΔ0-applied sections were incubated in a sealed vessel containing water at 32 °C for 1 h to allow the sections to adequately moisten and maximize the derivatization reaction with amino acids and amines. It enabled us to take many images of amino acids and amines derivatized by mTRAQΔ0 (Fig. 3). This result indicates that mTRAQΔ0 is compatible with a range of amino acids and amines.

### Confirmation of GABA derivatization

**Fig. 4:**
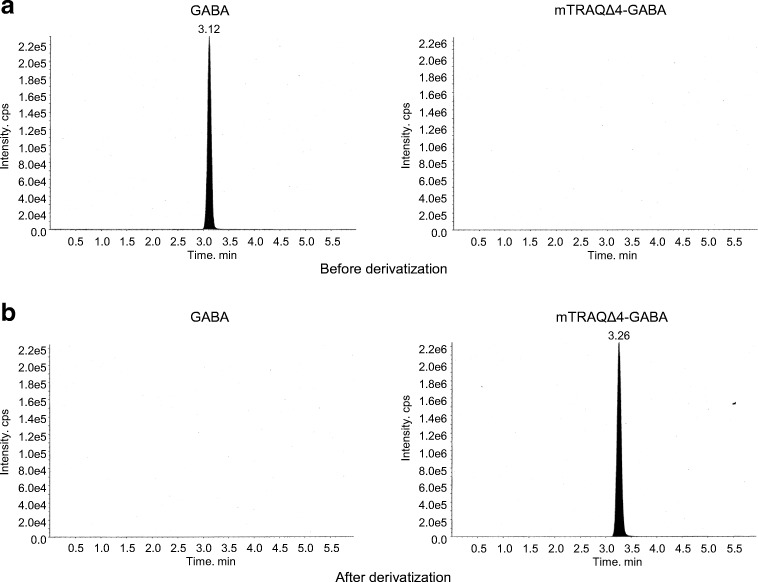
Confirmation of the GABA derivatization by LC-MS/MS used as internal standard for the triple spray. Chromatograms of GABA and mTRAQΔ4-GABA are from **a** before derivatization and **b** after derivatization. mTRAQΔ4-GABA, GABA labeled with mTRAQΔ4

The use of GABA labeled with mTRAQΔ4 as the internal standard solution is necessary for normalization, so we confirmed the degree of the derivatization by LC-MS/MS. mTRAQΔ4 reagent and TEAB buffer were added to the GABA standard solution and left to react for 2 h at room temperature. At the end of reaction period, hydroxylamine was added to eliminate any remaining mTRAQΔ4, thereby preventing reaction of mTRAQΔ4 with endogenous GABA in the tissue when the internal standard solution was sprayed onto the slice. To confirm the derivatization efficiency, we measured GABA and GABA labeled with mTRAQΔ4 before and after the derivatization step. As a result, GABA was completely eliminated after derivatization and GABA labeled with mTRAQΔ4 was successfully formed (Fig. 4).

### Reproducibility of GABA quantitation using triple spray method

**Fig. 5:**
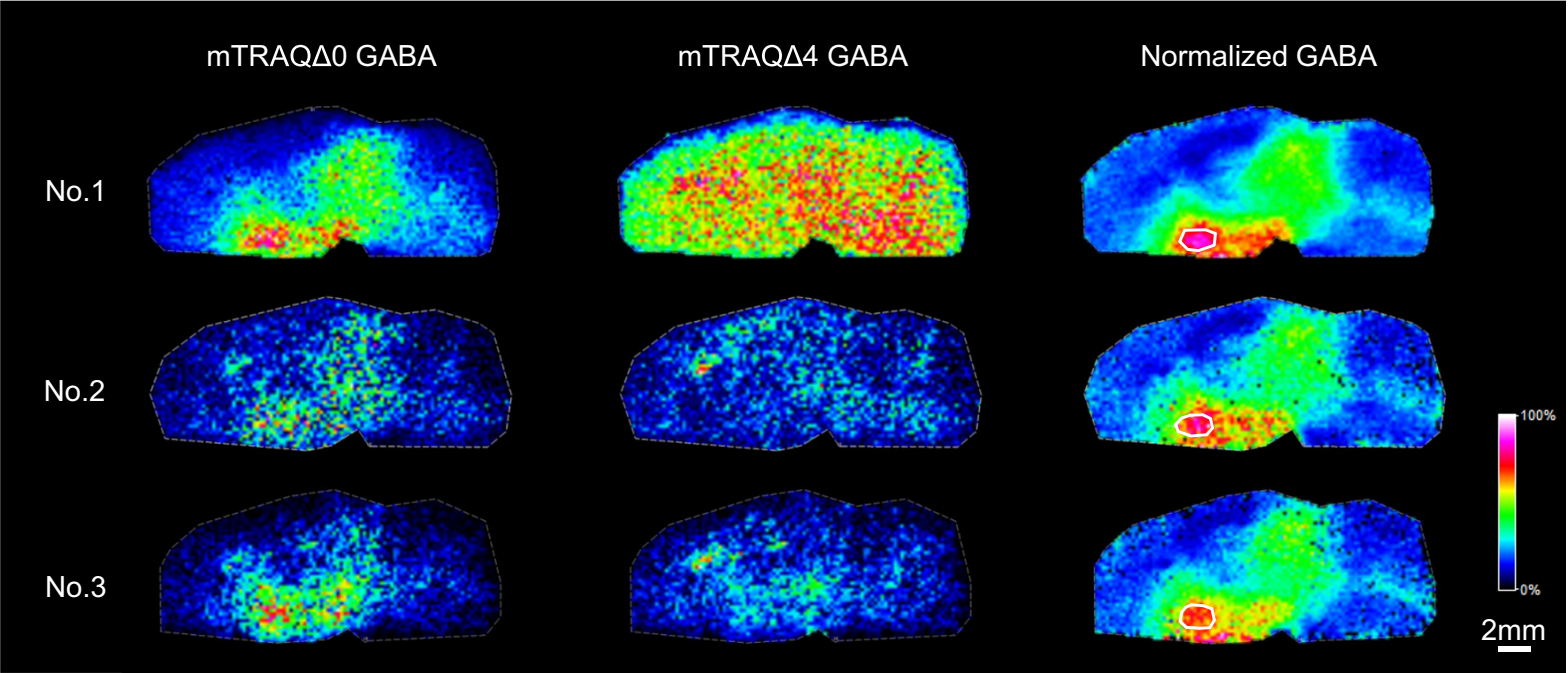
The reproducibility of triple spray GABA measurement using serial sections. The region of interest was set at the hypothalamus in each serial slice similarly, and the corresponding regions are marked with white frame in normalized GABA images. mTRAQΔ0-GABA, GABA labeled with mTRAQΔ0; mTRAQΔ4-GABA, GABA labeled with mTRAQΔ4; normalized GABA, corrected quantities of GABA calculated from the ratio of GABA labeled with mTRAQΔ0 to GABA labeled with mTRAQΔ4. Scale bar, 2 mm; spatial resolution = 200 μm

To confirm the reproducibility of the triple spray, we compared the GABA distribution in three serial sections of WKY rat brain by utilizing the optimized mTRAQ reaction conditions. We set the region of interest at the hypothalamus, a high GABA concentration area, and calculated the ratio of the average peak intensity of GABA labeled with mTRAQΔ0 to GABA labeled with mTRAQΔ4 (Fig. 5, Table 1). Our results showed a variation in the average peak intensity of GABA labeled with mTRAQΔ0 and GABA labeled with mTRAQΔ4 across the three serial sections, but the ratio calculated for each section showed a little variation, which confirmed the effectiveness of our internal standard normalization method.

**Table 1:** Comparison of the region of interest of three serial sections of WKY rat brain

Sample	mTRAQΔ0 GABA (peak intensity)	mTRAQΔ4 GABA (peak intensity)	Normalized GABA (ratio)	Mean	SD	CV (%)
No. 1	16,735,218	19,256,516	0.87	0.87	0.014	1.62
No. 2	3,815,740	4,323,374	0.88			
No. 3	6,270,675	7,339,209	0.85			

Ratio was calculated by peak intensities of mTRAQΔ0 GABA to mTRAQΔ4 GABA peak. Mean, SD, and CV (%) were calculated based on the three ratios

### Comparison of the GABA level in WKY and SHRSP rats

**Fig. 6:**
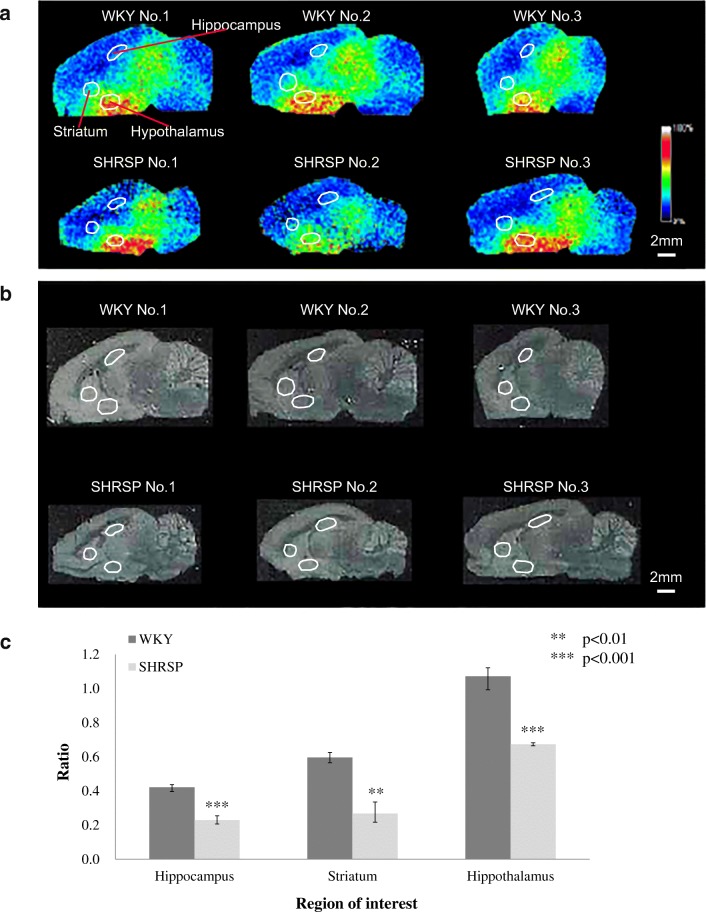
Comparison of normalized GABA distribution in WKY and SHRSP rats. Region of interest was set at the hippocampus, striatum, and hypothalamus in these rat brains similarly, and the corresponding regions are marked with white frame. **a** Mass spectrometry image. Scale bar, 2 mm; spatial resolution = 200 μm. **b** Optical image. **c** GABA concentration was compared using ratio in each part

From the above test, the reproducibility of the triple spray was confirmed, so we proceeded with the comparison of the animal models. We prepared three individuals each of SHRSP and WKY rats with the same week of age. SHRSP is a rat model conditioned with high blood pressure and made prone to strokes induced by providing salty food and water. In this study, SHRSP rats were given 1% saline for a week to induce them into a state immediately before causing a stroke because the areas affected by the stroke can differ among individuals, leading to a difficulty in cross-model comparison. In the analysis, we compared the GABA distribution at region of interest (Fig. 6, Table 2). In each site, the GABA ratio was lower in SHRSP than in WKY, which was consistent with previous studies. Also, our results show a small variation within the respective models but showed a significant difference between the models. From these results, it became clear that the triple spray can effectively evaluate different models.

**Table 2:** Comparison of three different brain regions of interest in WKY and SHRSP rats

	Hippocampus			Striatum			Hypothalamus		
	Peak intensity		Ratio	Peak intensity		Ratio	Peak intensity		Ratio
	mTRAQΔ0 GABA	mTRAQΔ4 GABA		mTRAQΔ4 GABA	mTRAQΔ4 GABA		mTRAQΔ0 GABA	mTRAQΔ4 GABA	
WKY no. 1	580,921	1,464,578	0.40	1,036,339	1,655,661	0.63	1,828,763	1,660,663	1.10
WKY no. 2	857,537	1,999,239	0.43	1,114,299	1,865,746	0.60	2,044,267	2,058,933	0.99
WKY no. 3	906,989	2,072,277	0.44	1,053,054	1,864,875	0.56	2,585,214	2,305,500	1.12
SHRSP no. 1	317,071	1,533,812	0.21	371,867	1,717,641	0.22	1,687,085	2,511,636	0.67
SHRSP no. 2	429,512	1,688,049	0.25	175,346	521,826	0.34	464,130	680,984	0.68
SHRSP no. 3	476,865	2,093,849	0.23	706,273	2,808,847	0.25	2,113,803	3,179,151	0.66
Region of interest		Ratio			CV (%)		*p* value		
		WKY	SHRSP		WKY	SHRSP			
Hippocampus		0.42 ± 0.02	0.23 ± 0.02		5.1	10.4	0.00050		
Striatum		0.60 ± 0.03	0.27 ± 0.06		5.1	22.9	0.00117		
Hypothalamus		1.07 ± 0.07	0.67 ± 0.01		6.4	1.2	0.00058		

Ratio value was calculated by peak intensities of mTRAQΔ0 GABA to mTRAQΔ4 GABA in each part and represented as mean ± SD (*n* = 3)

### Comprehensive analysis of water-soluble amino acids by triple spray method

**Fig. 7:**
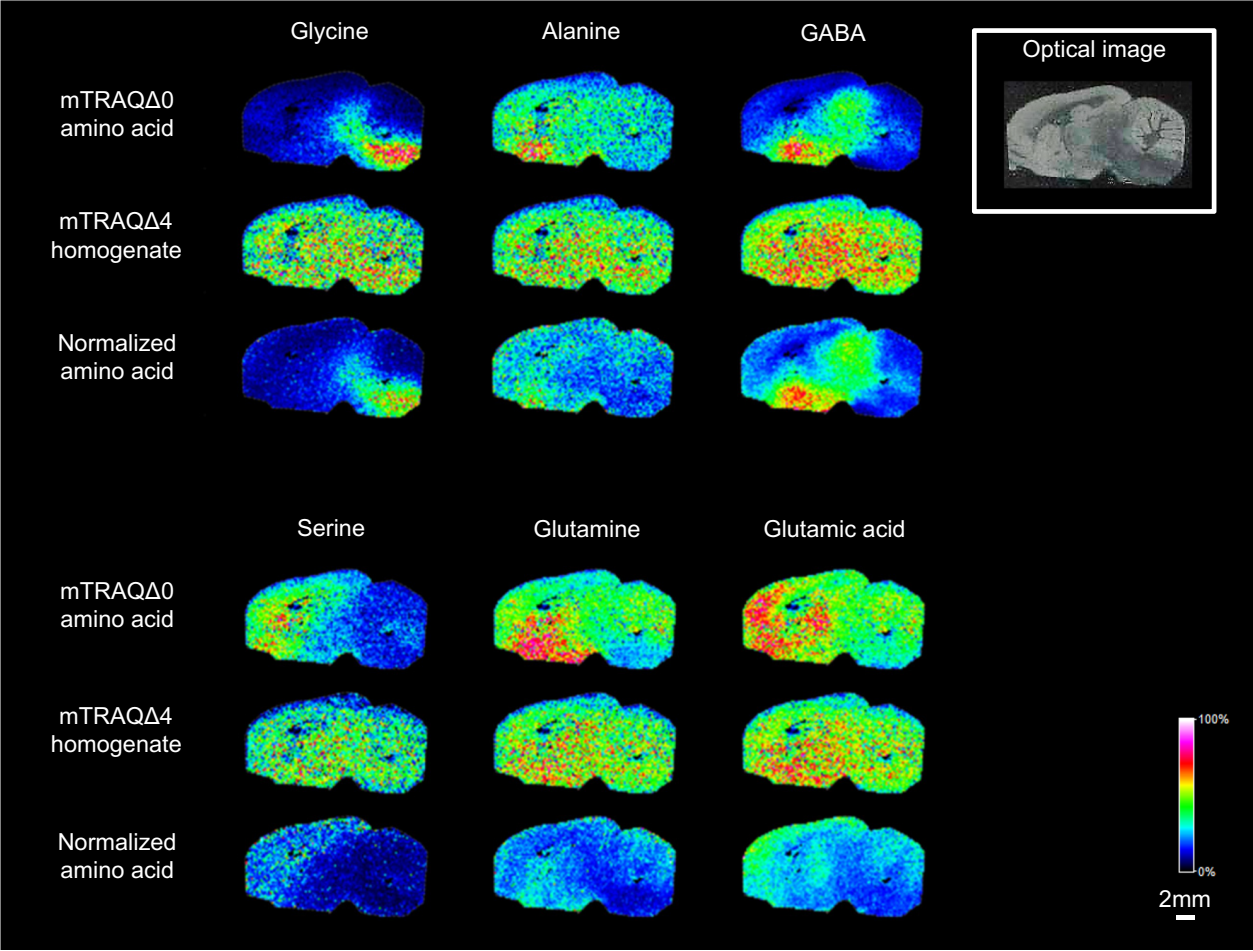
Comprehensive measurement of water-soluble amino acids. mTRAQΔ4 homogenate can normalize 6 amino acids at the same time. mTRAQΔ0 amino acid, each amino acid labeled with mTRAQΔ0; mTRAQΔ4 homogenate, water-soluble amino acids extracted from rat brain homogenate labeled with mTRAQΔ4; normalized amino acid, each amino acid labeled with mTRAQΔ0 to water-soluble amino acids extracted from rat brain homogenate labeled with mTRAQΔ4. Scale bar, 2 mm; spatial resolution = 200 μm

All the above tests for triple spray focused on one analyte. We explored the possibility of simultaneously measuring multiple analytes by conducting a comprehensive analysis of water-soluble amino acids. Since the triple spray method uses stable isotope–labeled derivatization reagent, it allows us to use water-soluble amino acids extracted from tissue homogenate as the internal standard by simply derivatizing them with the mTRAQ reagent. In this study, we used WKY rat brain prepared in the same three step spraying procedure. For the internal standard, we used the water-soluble amino acids extracted from rat brain homogenate by the Bligh and Dyer method [22] and derivatized with mTRAQΔ4. We succeeded in producing a simultaneously normalized image with regard to glycine, alanine, GABA, serine, glutamine, and glutamic acid (Fig. 7).

## Discussion

We tried to establish an analytical method to quantify amino acids and amines using stable isotope–labeled derivatization reagents through a relative comparison of the different slices. Derivatization is effective when analyzing low sensitivity molecule such as amino acids and amines, and numerous derivatization reagents have been reported. However, we thought of a normalization method utilizing the difference in molecular weight of the derivatization reagents. There are other experimentation methods, such as applying the analyte’s stable isotope first onto the slice following with the application of the derivatization reagents. With this method, only one type of derivatization reagent is necessary and will be able to account for the derivatization efficiency for each internal tissue. However, in order to conduct this experiment, it is vital to evenly apply the stable isotope across the entire tissue section in the first stage of spray application, which is difficult with automatic sprays currently available. Furthermore, this method would not allow for simultaneous analysis of several analytes, which led us to choose the triple spray method. We focused on mTRAQ reagents because they have not been reported for their application in MALDI mass spectrometry imaging and because of their ability to derivatize a range of amino acids and amines, making it suitable for the triple spray. In the reproducibility test, we observed varied GABA responses from each slice, but with normalization of data, we were able to show a comparable result between the slices. This showed that the triple spray method with the normalization calculation allowed for effective correction of variation caused by matrix solution spray application and change in instrumental sensitivity. This method is a relative quantification, not an absolute quantification. This is because of the problem in the derivatization efficiency and the use of one-point calibration. Therefore, the calculated values are only able to show the change in GABA levels. Incidentally, as for catecholamine, which is a group of important neurotransmitters, serotonin and norepinephrine could not be detected with on-tissue derivatization, while endogenous dopamine could be detected but its internal standard could not (data not shown). The cause is unclear but we think that mTRAQ might not be suitable for catecholamine derivatization. There are other derivatization reagents labeled with stable isotope [23], and with the possibility of each reagent working differently with individual amino acids and amines, we hope to further develop and expand the variation of detectable amino acids and amines.

For the comparison between the two animal models, we obtained a good result. The variation in the values for each model was around 2-fold with a small CV value. The ability to reliably produce variation within several tens of percentage is a promising sign when compared with the medical diagnosis practice using biomarkers, because they too are evaluated in the similar order of magnitude. We hope that this technique could be further developed to aid the understanding of pathology for animal models in drug development. GABA is an inhibitory neurotransmitter and its concentration has been reported to decrease after the onset of strokes. SHRSP is commonly used as an ischemic brain disease model because it is prone to cause stroke. So far, microdialysis has been used to measure neurotransmitter in the brain [24]. In this study, we compared the same specified site in the brain between SHRSP and WKY rats and found that GABA levels were lower in SHRSP than in WKY rats. This result was consistent with previous studies [17].

As for the measurement of water-soluble amines, we succeeded in normalizing 6 amino acids at the same time. The advantage of using tissue-derived internal standards is that the content of endogenous substances is the same as in the tissue slices, so we can more accurately estimate the concentration of the internal standard, making this an advantage in conducting unknown biomarker search. However, tissue homogenate includes many other substances, which could lead to ion suppression and increased viscosity of the solution, thereby raising the need to optimize the spraying conditions with different tissues. To solve these problems, we would need further studies.

From this study, it is clear that the triple spray can compare tissues reproducibly and this has the potential to be developed into a method which could account for the variation caused by ion suppression from the tissue. However, this method still has room for improvement. Amino acids and amines were exclusively used in this study, but mTRAQ reagents are most known for their use in derivatizing targeted proteins and peptides to aid LC/MS analysis. We hope to further develop the triple spray to be able to analyze proteins and peptides by investigating how each amino group behaves on tissue in the three-step spraying procedure. With further research, we hope to devise an improved method of quantification where the triple spray will become a new effective research tool of drug development.

## References

[1] Schulz S, Becker M, Groseclose MR, Schadt S, Hopf C (2019). Advanced MALDI mass spectrometry imaging in pharmaceutical research and drug development. Curr Opin Biotechnol.

[2] Bednarczyk K, Gawin M, Chekan M, Kurczyk A, Mrukwa G, Pietrowska M, Polanska J, Widlak P (2019). Discrimination of normal oral mucosa from oral cancer by mass spectrometry imaging of proteins and lipids. J Mol Histol.

[3] Kashimura A, Tanaka K, Sato H, Kaji H, Tanaka M (2018). Imaging mass spectrometry for toxicity assessment: a useful technique to confirm drug distribution in histologically confirmed lesions. J Toxicol Pathol.

[4] Calvano CD, Monopoli A, Cataldi TRI, Palmisano F (2018). MALDI matrices for low molecular weight compounds: an endless story?. Anal Bioanal Chem.

[5] Esteve C, Tolner EA, Shyti R, van den Maagdenberg AMJM, McDonnell LA (2016). Mass spectrometry imaging of amino neurotransmitters: a comparison of derivatization methods and application in mouse brain tissue. Metabolomics.

[6] Quanico J, Franck J, Wisztorski M, Salzet M, Fournier I, Kobeissy FH, Stevens SM (2017). Progress and potential of imaging mass spectrometry applied to biomarker discovery. Neuroproteomics.

[7] Nakashima Y, Setou M (2018). Distribution of antisense oligonucleotides in rat eyeballs using MALDI imaging mass spectrometry. Mass Spectrom.

[8] Rao T, Shen B, Zhu Z, Shao Y, Kang D, Li X, Yin X, Li H, Xie L, Wang G, Liang Y (2017). Optimization and evaluation of MALDI TOF mass spectrometric imaging for quantification of orally dosed octreotide in mouse tissues. Talanta.

[9] Groseclose MR, Castellino S (2013). A mimetic tissue model for the quantification of drug distributions by MALDI imaging mass spectrometry. Anal Chem.

[10] Gemperline E, Rawson S, Li L (2014). Optimization and comparison of multiple MALDI matrix application methods for small molecule mass spectrometric imaging. Anal Chem.

[11] Reich RF, Cudzilo K, Levisky JA, Yost RA (2010). Quantitative MALDI-MS n analysis of cocaine in the autopsied brain of a human cocaine user employing a wide isolation window and internal standards. J Am Soc Mass Spectrom.

[12] Bergman H-M, Lundin E, Andersson M, Lanekoff I (2016). Quantitative mass spectrometry imaging of small-molecule neurotransmitters in rat brain tissue sections using nanospray desorption electrospray ionization. Analyst.

[13] Toue S, Sugiura Y, Kubo A, Ohmura M, Karakawa S, Mizukoshi T, Yoneda J, Miyano H, Noguchi Y, Kobayashi T, Kabe Y, Suematsu M (2014). Microscopic imaging mass spectrometry assisted by on-tissue chemical derivatization for visualizing multiple amino acids in human colon cancer xenografts: Proteomics 2014. PROTEOMICS.

[14] Manier ML, Spraggins JM, Reyzer ML, Norris JL, Caprioli RM (2014). A derivatization and validation strategy for determining the spatial localization of endogenous amine metabolites in tissues using MALDI imaging mass spectrometry: endogenous amine metabolites in tissue. J Mass Spectrom.

[15] Yeom J, Kang MJ, Shin D, Song HK, Lee C, Lee JE (2015). mTRAQ-based quantitative analysis combined with peptide fractionation based on cysteinyl peptide enrichment. Anal Biochem.

[16] Shariatgorji M, Nilsson A, Goodwin RJA, Källback P, Schintu N, Zhang X, Crossman AR, Bezard E, Svenningsson P, Andren PE (2014). Direct targeted quantitative molecular imaging of neurotransmitters in brain tissue sections. Neuron.

[17] Tuomisto L, Yamatodani A, Dietl H, Waldmann U, Philippu A (1983). In vivo release of endogenous catecholamines, histamine and GABA in the hypothalamus of Wistar Kyoto and spontaneously hypertensive rats. Naunyn Schmiedeberg's Arch Pharmacol.

[18] Cao Q, Wang Y, Chen B, Ma F, Hao L, Li G, Ouyang C, Li L (2019). Visualization and identification of neurotransmitters in crustacean brain via multifaceted mass spectrometric approaches. ACS Chem Neurosci.

[19] Enomoto Y, Nt. An P, Yamaguchi M, Fukusaki E, Shimma S (2018). Mass spectrometric imaging of GABA in the *Drosophila melanogaster* adult head. Anal Sci.

[20] Shirran SL, Botting CH (2010). A comparison of the accuracy of iTRAQ quantification by nLC-ESI MSMS and nLC-MALDI MSMS methods. J Proteome.

[21] Cobice DF, Livingstone DEW, Mackay CL, Goodwin RJA, Smith LB, Walker BR, Andrew R (2016). Spatial localization and quantitation of androgens in mouse testis by mass spectrometry imaging. Anal Chem.

[22] Fei F, Bowdish DME, McCarry BE (2014). Comprehensive and simultaneous coverage of lipid and polar metabolites for endogenous cellular metabolomics using HILIC-TOF-MS. Anal Bioanal Chem.

[23] Tatsuta Y, Tanaka Y, Ikeda A, Matsukawa S, Katano H, Taira S (2017). Nanoparticle-assisted laser desorption/ionization mass spectrometry (Nano-PALDI MS) with Py-Tag for the analysis of small molecules. Mass Spectrom.

[24] Kehr J, Yoshitake T, Ichinose F, Yoshitake S, Kiss B, Gyertyán I, Adham N (2018). Effects of cariprazine on extracellular levels of glutamate, GABA, dopamine, noradrenaline and serotonin in the medial prefrontal cortex in the rat phencyclidine model of schizophrenia studied by microdialysis and simultaneous recordings of locomotor activity. Psychopharmacology.

